# Supplementary material for: School Entry Deferral: Effects of Age and Schooling on Children's Longitudinal Executive Function Development and Parent‐Reported Socio‐Emotional and Regulatory Outcomes
Source: Dev Sci. 2026 Jul 21;29(5):e70257. doi: 10.1111/desc.70257 (PMC13386192; doi:10.1111/desc.70257)
Supplement: Supplementary file 1 — Supporting Information: desc70257‐supp‐0001‐SuppMat.docx [file DESC-29-e70257-s001.docx]

# Supplementary Material

**Table S.1**

Demographic and outcome comparison between participants who took part at both assessment points (retained) and those who dropped out of the study at T2 (drop-out)

|  | |  | Sample | |  | | | |  |
| --- | --- | --- | --- | --- | --- | --- | --- | --- | --- |
|  | |  | Retained (T1&T2) | Drop-out (T1 only) | | Chi-square | | | |
|  | |  | *n* | *n* | | *χ^2^(1)* | *p* | *Cramer’s V* | |
|  | *n* | | 110 | 8 | |  |  |  | |
| Gender | female | | 52 | 6 | | 1.49 | .222 | .112 | |
|  | male | | 58 | 2 | |  |  |  |  |
| Neighbourhood deprivation  (SIMD Quintiles) | 1-3 | | 34 | 4 | | 1.20 | .273 | .101 | |
|  | 4-5 | | 75 | 4 | |  |  |  |  |
|  |  | |  |  | |  |  |  |  |
|  | |  |  |  | | Mann-Whitney U Test | | | |
|  | |  | *Mean (sd)* | *Mean (sd)* | | *Z* | *p* | *r* | |
| T1 Inhibitory Control | |  | 2167 (695) | 2476 (1135) | | -0.59 | .559 | -0.05 | |
| T1 Cognitive Flexibility | |  | 2544 (806) | 2213 (413) | | 0.84 | .404 | 0.08 | |
| T1 Working Memory | |  | 15.49 (7.85) | 10.00 (8.83) | | 1.98* | .048 | 0.18 | |
| T1 Vocabulary ^a^ | |  | 12.22 (2.44) | 9.25 (1.91) | | 3.03** | .002 | 0.28 | |
| T1 Nonverbal Reasoning ^a^ | |  | 12.09 (2.63) | 9.75 (3.06) | | 2.05* | .040 | 0.19 | |
| T1 Socialising | |  | 4.17 (0.57) | 4.08 (0.72) | | 0.24 | .808 | 0.02 | |
| T1 Prosocial Behaviour | |  | 4.18 (0.48) | 4.23 (0.21) | | -0.27 | .789 | -0.03 | |
| T1 Behavioural Self-regulation | |  | 4.00 (0.49) | 4.12 (0.42) | | -0.56 | .573 | -0.05 | |
| T1 Cognitive Self-regulation | |  | 3.74 (0.55) | 4.09 (0.51) | | -1.45 | .147 | -0.14 | |
| T1 Emotional Self-regulation | |  | 3.57 (0.71) | 3.62 (0.37) | | 0.01 | .995 | 0.00 | |
| T1 Externalising | |  | 2.00 (0.63) | 1.91 (0.46) | | 0.32 | .748 | 0.03 | |

*Note*. ^a^ Age-standardised Scores. Significance: *p* < .01**, *p* < .05* Effect size (*r*): small = 0.10, medium = 0.24, large = 0.38).

**Table S.2**

*Summary of missing data within the final sample & reason for exclusion.*

|  |  | Def PreS | | Non-def P1 | | Def P1 | |
| --- | --- | --- | --- | --- | --- | --- | --- |
| Measure | Reason | T1 | T2 | T1 | T2 | T1 | T2 |
| Flanker | Task completed using Tablet |  | 1 |  |  | 2 | 1 |
|  | Low accuracy (≤50%) |  | 1 |  |  |  |  |
| DCCS Single Block | Task completed using Tablet |  | 1 |  |  | 2 | 1 |
|  | Computer rebooted | 1 |  |  |  |  |  |
|  | Insufficient trials (≤20%) / Low accuracy (≤50%) | 1 | 1 |  |  |  |  |
|  | Fatigue | 1 |  |  |  |  |  |
| DCCS Mixed Block | Task completed using Tablet |  | 1 |  |  | 2 | 1 |
|  | Fatigue | 5 | 1 |  |  | 1 |  |
|  | Computer rebooted | 1 |  |  |  |  |  |
|  | Low accuracy (≤50%) | 3 | 1 |  | 1 |  | 1 |
| Animal Race | Did not understand task |  |  | 1 |  | 2 |  |
|  | Did not attend session |  |  | 1 |  |  |  |
| Picture Naming | Did not attend session |  |  | 1 |  |  |  |
|  | Fatigue | 1 |  |  |  |  |  |
| Matrix Reasoning | Did not attend session |  |  | 1 |  |  |  |
|  | Fatigue | 1 |  |  |  |  |  |
| CSBQ | Suspected/diagnosed ASD |  |  |  |  | 2 | 2 |
|  | Parent non-completion |  | 1 | 1 |  |  | 1 |
|  | Outlying scores | 1 | 1 | 1 | 1 |  |  |

*Note*. ASD = Autism Spectrum Disorder. Outlying scores (±3sd) were calculated at the group level.

**Table S.3**

*Alpha values reflecting the internal consistency in the CSBQ subscales at T1 and T2*

|  | T1 | T2 |
| --- | --- | --- |
| Subscale | α | α |
| Externalising | 0.73 | 0.71 |
| Internalising | 0.46 | 0.46 |
| Socialising | 0.83 | 0.77 |
| Prosocial Behaviour | 0.80 | 0.80 |
| Cognitive Self-Regulation | 0.70 | 0.76 |
| Behavioural Self-Regulation | 0.65 | 0.74 |
| Emotional Self-Regulation | 0.80 | 0.73 |

**Table S.4**

*Number and percentage of trials removed during data preprocessing.*

|  |  | Assessed trials | | | |
| --- | --- | --- | --- | --- | --- |
|  |  | Total trials | Trials removed | | |
|  |  | *n* | Reason for removal | *n* | Percent of trials |
| Flanker | T1 | 5184 | RT < 200ms | 37 | 0.71% |
|  |  |  | RT > 10sec | 95 | 1.83% |
|  |  |  | RT outlier | 125 | 2.63% ^Ϯ^ |
|  | T2 | 5184 | RT < 200ms | 17 | 0.33% |
|  |  |  | RT > 10sec | 47 | 0.91% |
|  |  |  | RT outlier | 120 | 2.43% ^Ϯ^ |
| DCCS | T1 | 4176 | RT < 200ms | 49 | 1.17% |
|  |  |  | RT > 10sec | 112 | 2.68% |
|  |  |  | RT outlier | 18 | 0.50% ^Ϯ^ |
|  | T2 | 4256 | RT < 200ms | 36 | 0.85% |
|  |  |  | RT > 10sec | 59 | 1.39% |
|  |  |  | RT outlier | 16 | 0.42% ^Ϯ^ |

^Ϯ^ Percent of accurate trials

**Figure S.1**

*Histogram of z-scores for baseline age (T1) among the Non-deferred P1(green) and Deferred Preschool group (blue).*


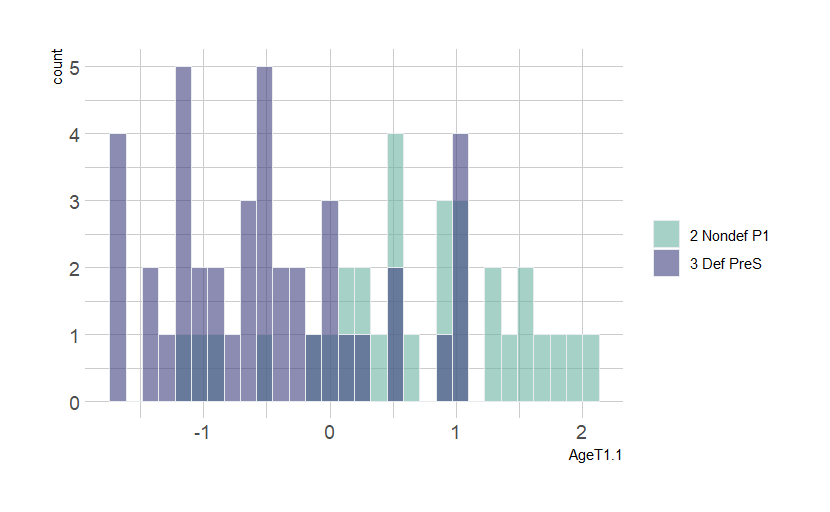


*n*

**Group**

Non-deferred P1

Deferred Preschool

Relative age (z-score)

*Note.* A z-score of 0 reflect the average age of the participants in this comparison, with a score of ±1 associated with children who were on average 64 days younger/older than the average (84% of Non-deferred P1s were older than the average, while 79% of Deferred Pre-schoolers were younger than the average age).

**Figure S.2**

*Histogram of z-scores for baseline (T1) assessment date of the Deferred P1 group (green) and the Non-deferred P1 group (blue). Solid black line references the beginning of the academic year.*


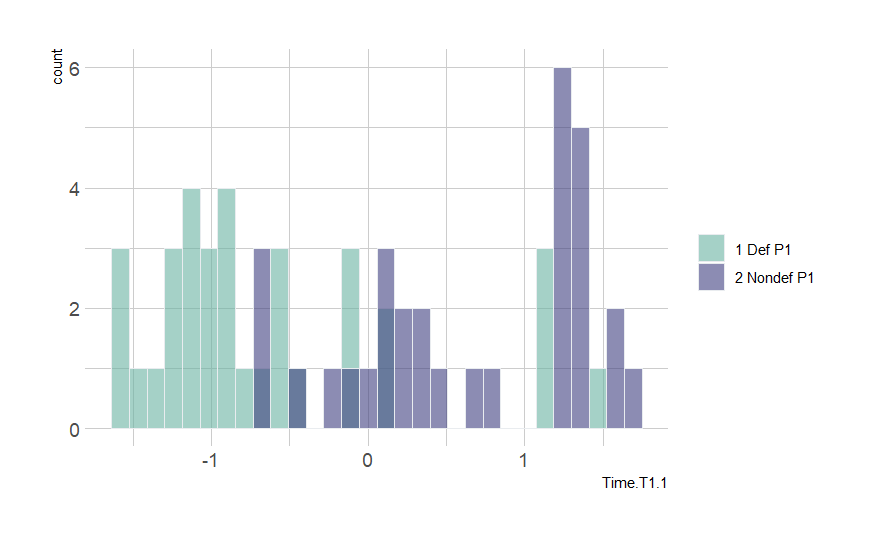


*n*

Date of assessment (z-score)

**Group**

Deferred P1

Non-deferred P1

*Note.* A z-score of 0 indicates the mid-point of our baseline assessment (Day 81.5), and 1 standard deviation was equivalent to ± 50.27 days (a score of -1 would reflect assessment on Day 31 of this period, while a score of 1 would represent assessment on Day 131).

**Figure S.3**

*Violin plot of group-level EF outcomes, including participant-level scores between testing points (left) and change scores (right).*

*
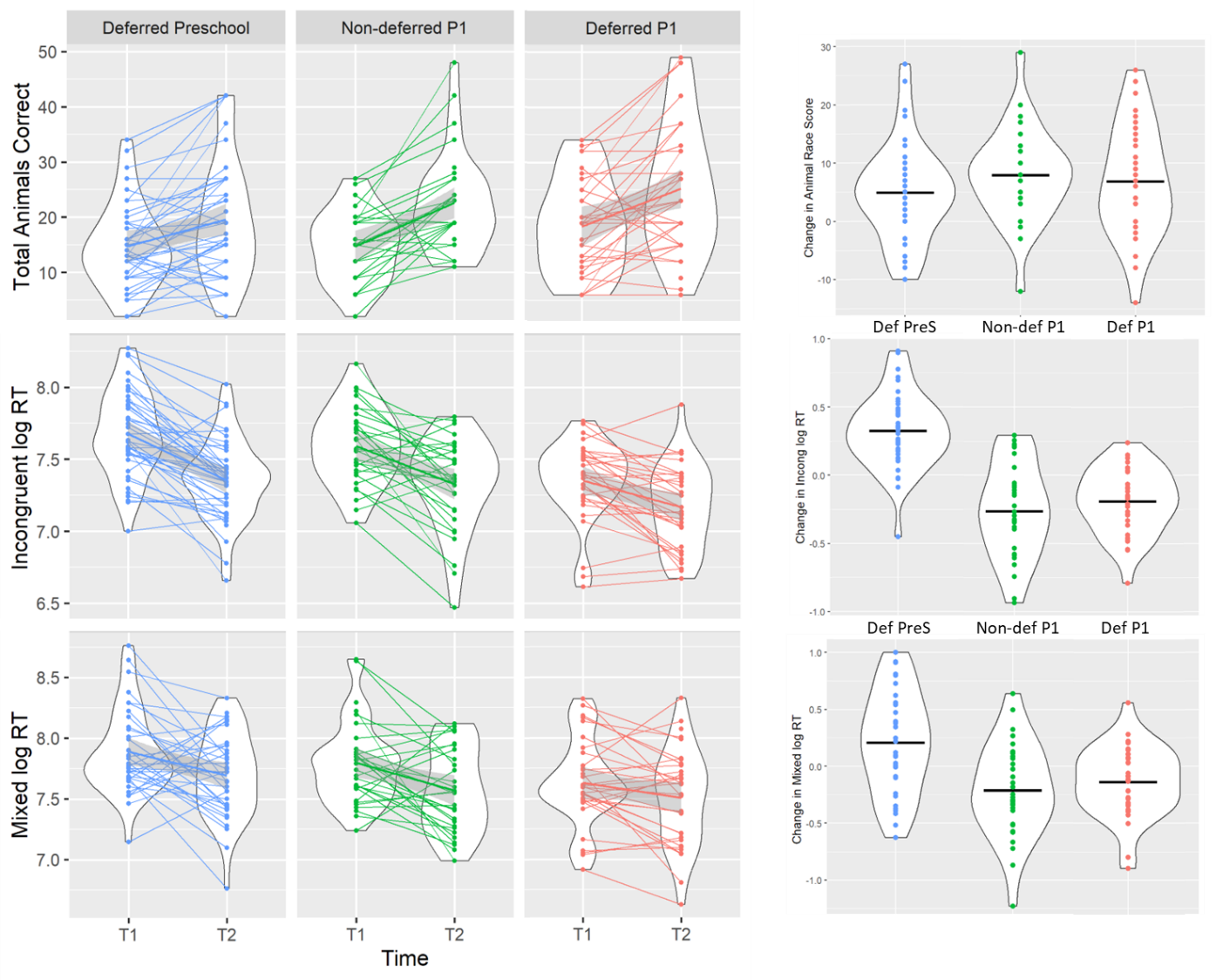
*

*
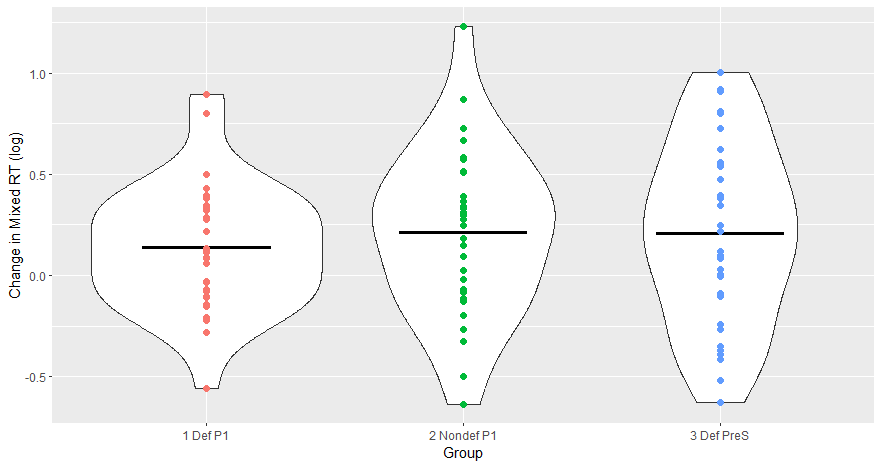
*

**Figure S.4**

*Violin plot of group-level CSBQ subscales, including participant-level scores between testing points (left) and change scores (right).*


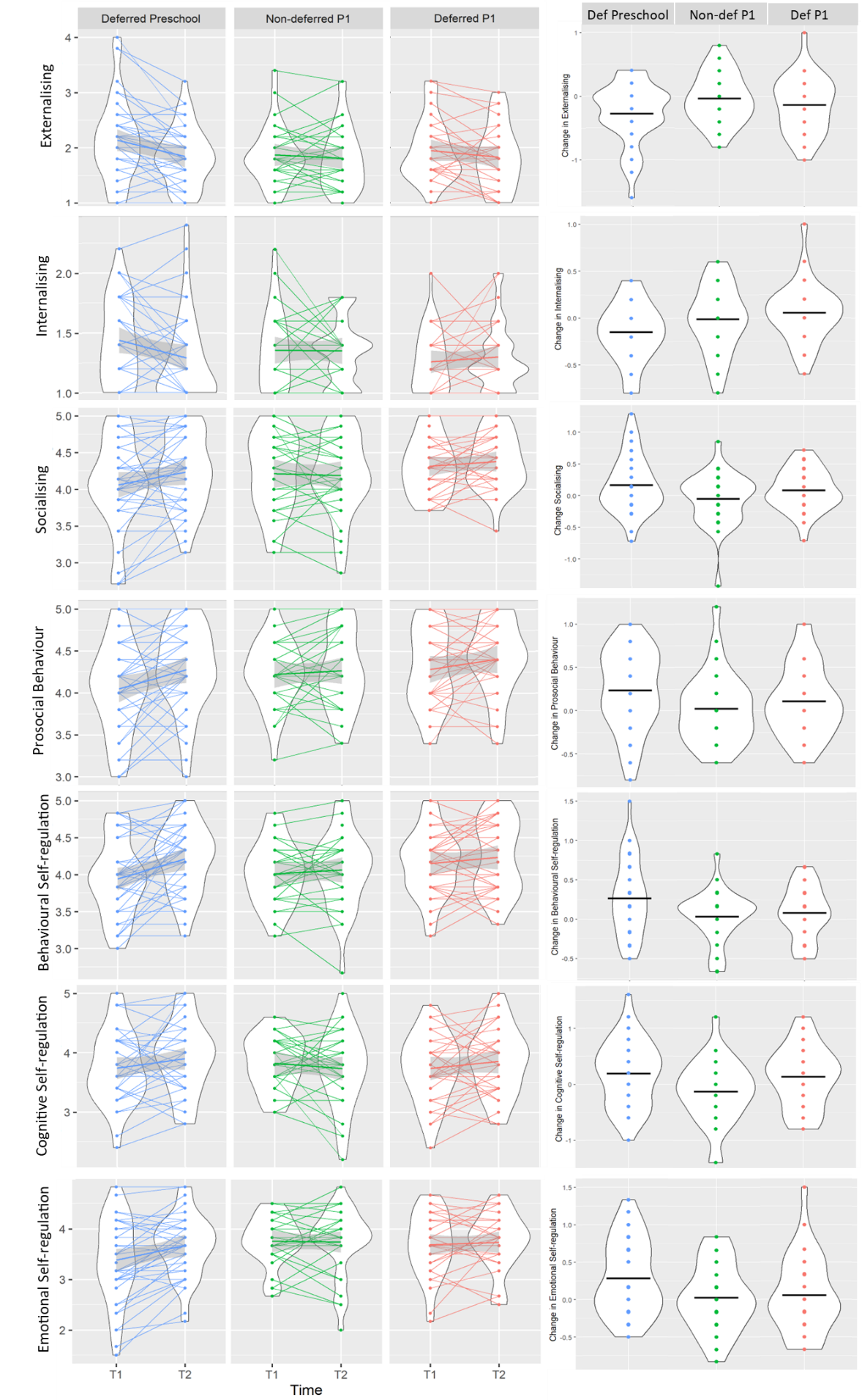


**Table S.5**

Spearman’s correlation coefficients for associations between cognitive outcomes (pooled across groups), within- (grey) and across-domains.

|  | Inhibitory Control (Incongruent log RT) | | |  | Shifting  (Mixed log RT) | | |  | Working Memory  (Animals Correct) | | |
| --- | --- | --- | --- | --- | --- | --- | --- | --- | --- | --- | --- |
|  | T1 | T2 | Change |  | T1 | T2 | Change |  | T1 | T2 | Change |
| T1 Vocabulary ^a^ | -.219* | -.273** | -.029 |  | -.338** | -.277** | .045 |  | .264** | .342** | -.041 |
|  |  |  |  |  |  |  |  |  |  |  |  |
| T1 Nonverbal Reasoning ^a^ | -.347** | -.254** | .056 |  | -.324** | -.240** | -.020 |  | .094 | .125 | .091 |
|  |  |  |  |  |  |  |  |  |  |  |  |
| T1 Inhibitory Control |  | .526** | -.484** |  | .526** | .302** | -.205* |  | -.240* | -.237* | -.041 |
|  |  |  |  |  |  |  |  |  |  |  |  |
| T1 Shifting | .526** | 430** | -.140 |  |  | .397** | -.503** |  | -.290** | -.207* | -.033 |
|  |  |  |  |  |  |  |  |  |  |  |  |
| T1 Working Memory | -.240* | -.380** | -.144 |  | -.290** | -.256* | .017 |  |  | .570** | -.149 |

*Note*. Change scores are calculated as T2 less T1 scores. A smaller change score on RT outcomes reflects a larger improvement/change over time.

^a^ Raw Scores. Significance: *p* < .01**, *p* < .05*

**Table S.6**

Spearman’s correlation coefficients for associations between CSBQ outcomes (pooled across groups), within- (grey) and across-domains.

|  |  | T1 Externalising | T1  Socialising | T1  Prosocial Behaviour | T1 Behavioural Self-reg | T1  Cognitive Self-reg | T1  Emotional Self-reg |
| --- | --- | --- | --- | --- | --- | --- | --- |
| Externalising | T1 |  | -.115 | -.487** | -.388** | -.278** | -.819** |
|  | T2 | .712** | -.041 | -.380** | -.433** | -.120 | -.569** |
|  | Change | -.432** | .050 | .172 | -.011 | .218* | .391** |
| Socialising | T1 | -.115 |  | .497** | .097 | .198* | .127 |
|  | T2 | -.128 | .666** | .299** | .095 | .161 | .156 |
|  | Change | -.056 | -.447** | -.244* | -.074 | -.071 | .005 |
| Prosocial Behaviour | T1 | -.487** | .497** |  | .555** | . 410** | .439** |
|  | T2 | -.312** | .377** | .594* | .450** | .307** | .347** |
|  | Change | .195* | -.158 | -.464** | -.164 | -.183 | -.113 |
| Behavioural Self-Regulation | T1 | -.388** | .097 | .555** |  | .378** | .358** |
|  | T2 | -.445** | .096 | .454** | .649** | .322** | .388** |
|  | Change | -.017 | .049 | -.094 | -.335** | -.090 | -.000 |
| Cognitive  Self-Regulation | T1 | -.278** | .198* | .410** | .378** |  | .285** |
|  | T2 | .048 | .143 | .280** | .247** | .480** | -.048 |
|  | Change | .335** | -.065 | -.109* | -.155 | -.448** | -.337* |
| Emotional  Self-regulation | T1 | -.819** | .127 | .439** | .358** | .285** |  |
|  | T2 | -.629** | .033 | .314** | .361** | .188 | .714** |
|  | Change | .401** | .169 | -.263** | -.102 | -.197* | -.515** |

*Note*. Change scores are calculated as T2 less T1. A smaller change score on externalising and internalising reflects a larger improvement/change over time. Significance: *p* < .01**, *p* < .05*

**Table S.7**

PCA loadings for the CSBQ composite at T1 and T2

|  | Principal Component | |
| --- | --- | --- |
| Subscale | CSBQ T1 | CSBQ T2 |
| Prosocial Behaviour | 0.81 | 0.85 |
| Socialising | 0.39 | 0.51 |
| Behavioural Self-regulation | 0.72 | 0.79 |
| Cognitive Self-regulation | 0.51 | 0.45 |
| Emotional Self-regulation | 0.82 | 0.76 |
| Externalising | -0.80 | -0.78 |
|  |  |  |
| Sum of squared loadings | 2.90 | 2.99 |
| Proportion of variance | 0.48 | 0.50 |
|  |  |  |

**Table S.8**

Parameter estimates (se) for the univariate latent change scores models for Flanker Congruent trials and DCCS Single Block trials within the schooling and age contrasts; and change in chi-square values and relative Akaike weights following parameter constraint (grey)

|  | Schooling Contrast | | | | | | | | |  | Age Contrast | | | | | | | | |
| --- | --- | --- | --- | --- | --- | --- | --- | --- | --- | --- | --- | --- | --- | --- | --- | --- | --- | --- | --- |
|  | Flanker Congruent Log RT | | | |  | DCCS Single Block Log RT | | | |  | Flanker Congruent Log RT | | | |  | DCCS Single Block Log RT | | | |
|  | Def  PreS | Non-def  P1 | Likelihood  ratio test | |  | Def  PreS | Non-def  P1 | Likelihood  ratio test | |  | Non-def  P1 | Def  P1 | Likelihood ratio test | |  | Non-def  P1 | Def  P1 | Likelihood  ratio test | |
|  | *B (se)* | *B (se)* | *Δχ2 (1)* | *Rel w*(AIC) |  | *B (se)* | *B (se)* | *Δχ2 (1)* | *Rel w*(AIC) |  | *B (se)* | *B (se)* | *Δχ2 (1)* | *Rel w*(AIC) |  | *B (se)* | *B (se)* | *Δχ2 (1)* | *Rel w*(AIC) |
| T1 scores | 7.52 (0.04) | 7.47  (0.08) | 0.35 | 0.46 |  | 7.61 (0.07) | 7.51  (0.07) | 1.37 | 0.66 |  | 7.47 (0.08) | 7.35 (0.06) | 1.19 | 0.87 |  | 7.49  (0.07) | 7.39 (0.06) | 1.07 | 0.60 |
|  |  |  |  |  |  |  |  |  |  |  |  |  |  |  |  |  |  |  |  |
| Variance in T1 scores | 0.06** (0.01) | 0.07** (0.02) | 0.14 | 0.40 |  | 0.12** (0.03) | 0.11** (0.03) | 0.06 | 0.38 |  | 0.07** (0.02) | 0.08** (0.02) | 0.05 | 0.38 |  | 0.10** (0.03) | 0.08** (0.02) | 0.57 | 0.49 |
|  |  |  |  |  |  |  |  |  |  |  |  |  |  |  |  |  |  |  |  |
| Change (Δ) score | 4.14** (1.39) | 2.85* (1.39) | 0.34 | 0.44 |  | 5.09** (1.07) | 2.47* (1.22) | 3.32 | 1.07 |  | 2.80 (1.49) | 3.51** (1.23) | 0.15 | 0.39 |  | 2.60* (1.18) | 2.97* (1.51) | 0.04 | 0.37 |
|  |  |  |  |  |  |  |  |  |  |  |  |  |  |  |  |  |  |  |  |
| Variance in Δ score | 0.07** (0.02) | 0.09** (0.02) | 0.47 | 0.47 |  | 0.11**  (0.03) | 0.11** (0.03) | 0.01 | 0.37 |  | 0.09** (0.02) | 0.09** (0.03) | 0.00 | 0.37 |  | 0.11** (0.03) | 0.09** (0.03) | 0.15 | 0.42 |
|  |  |  |  |  |  |  |  |  |  |  |  |  |  |  |  |  |  |  |  |
| T1-Δ score Regression | -0.59** (0.19) | -0.42* (0.18) | 0.36 | 0.44 |  | -0.70** (0.14) | -0.35* (0.16) | 3.38 | 1.08 |  | -0.41* (0.20) | -0.51** (0.17) | 0.17 | 0.40 |  | -0.37* (0.16) | -0.42* (0.20) | 0.04 | 0.37 |
|  |  |  |  |  |  |  |  |  |  |  |  |  |  |  |  |  |  |  |  |
| Covariate^a^ onto T1 scores | 0.00 (0.04) | 0.03 (0.07) |  |  |  | -0.03 (0.05) | 0.06  (0.07) |  |  |  | 0.04 (0.07) | 0.08 (0.05) |  |  |  | 0.09 (0.09) | 0.04 (0.05) |  |  |
|  |  |  |  |  |  |  |  |  |  |  |  |  |  |  |  |  |  |  |  |
| Covariate^a^-  Δ score covariance | -0.05 (0.03) | 0.06 (0.05) |  |  |  | 0.03 (0.08) | 0.05  (0.05) |  |  |  | 0.04 (0.04) | -0.04 (0.05) |  |  |  | 0.05 (0.04) | -0.01 (0.04) |  |  |
|  |  |  |  |  |  |  |  |  |  |  |  |  |  |  |  |  |  |  |  |

*Note.*  Def PreS = Deferred Preschool group, Non-def P1 = Non-deferred P1 group, Def P1 = Deferred P1 group, Grp Diff = Group Difference.

Significance: *p < .05, **p < .01. ^a^ covariate: School Comparison = T1 Relative Age (z-score); Age Comparison = Time at T1 testing (z-score).

**Table S.9**

*Paired comparisons of the Congruency Effect (above) and Global Cost (below) broken down by time and group.*

|  | T1 | | | | |  | T2 | | | | | | | |  |
| --- | --- | --- | --- | --- | --- | --- | --- | --- | --- | --- | --- | --- | --- | --- | --- |
| Flanker | Congruent | Incongruent | Congruency Effect | Pairwise comparsion  (congruent vs incongruent RT) | |  | Congruent | Incongruent | | Congruency Effect | | Pairwise comparsion  (congruent vs incongruent RT) | | |  |
| Group | *ms* | *ms* | *ms* | *t(df)* | *95% CI* |  | *ms* | | *ms* | | *ms* | | *t(df)* | *95% CI* | |
| All | 1936 | 2159 | 223 | *t* (107) = -5.69** | [-300, -145] |  | 1483 | | 1602 | | 119 | | *t* (106) = -6.66** | [-153, -83] | |
| Deferred Preschool | 2086 | 2452 | 366 | *t* (43) = -5.16** | [-509, -223] |  | 1560 | | 1705 | | 145 | | *t* (41) = -4.72** | [-207, -83] | |
| Non-deferred P1 | 2015 | 2212 | 197 | *t* (30) = -2.97** | [-332, -61] |  | 1573 | | 1669 | | 96 | | *t* (30) = -3.05** | [-161, -31] | |
| Deferred P1 | 1663 | 1720 | 57 | *t* (32) = -1.21 | [-152, 38] |  | 1309 | | 1415 | | 106 | | *t* (33) = -3.58** | [-166, -45] | |
|  |  |  |  |  |  |  |  | |  | |  | |  |  | |
| DCCS | Single Blocks | Mixed Block | Global  Cost | Pairwise comparsion  (single vs mixed block RT) | |  | Single Blocks | | Mixed Block | | Global  Cost | | Pairwise comparsion  (single vs mixed block RT) | | |
| Group | *ms* | *ms* | *ms* | *t(df)* | *95% CI* |  | *ms* | | *ms* | | *ms* | | *t(df)* | *95% CI* | |
| All | 2141 | 2762 | 621 | *t* (97) = -6.51** | [-830, -442] |  | 1765 | | 2270 | | 505 | | *t* (101) = -8.74** | [-655, -413] | |
| Deferred Preschool | 2350 | 3123 | 773 | *t* (34) = -4.05** | [-1176, -390] |  | 1819 | | 2482 | | 663 | | *t* (38) = -7.34** | [-904, -513] | |
| Non-deferred P1 | 2214 | 2747 | 533 | *t* (30) = -4.13** | [-796, -269] |  | 1836 | | 2200 | | 364 | | *t* (29) = -3.40** | [-577, -143] | |
| Deferred P1 | 1817 | 2382 | 565 | *t* (31) = -3.32** | [-928, -222] |  | 1637 | | 2079 | | 442 | | *t* (32) = -4.42** | [-710, -262] | |
